# Intraoperative Laryngeal Mask Airway-Related Hiccup: An Overview

**DOI:** 10.31480/2330-4871/103

**Published:** 2019-10-14

**Authors:** Johann Mathew, Shiqian Shen, Henry Liu

**Affiliations:** 1 Department of Anesthesiology, Temple University Hospital, 3401 N Broad Street, Philadelphia, PA 19140, USA; 2 Department of Anesthesia, Critical Care & Pain Medicine, Massachusetts General Hospital, 55 Fruit Street, Boston, MA 02114, USA; 3 Department of Anesthesiology, Drexel University College of Medicine, Reading Hospital/Tower Health System, 420 S 5th Avenue, West Reading, PA 19611, USA

**Keywords:** Hiccup, Singultus, Laryngeal mask airway, iGel, COPA

## Abstract

Hiccup is an involuntary contraction of the diaphragm and intercostal muscles resulting in sudden inspiration and closure of the glottis. The presence of hiccup in the perioperative period can be a challenging problem. Sudden movements of the patient from hiccups can interfere preoperative diagnostic procedures, intraoperative hiccup may delay the beginning of surgery, interfere with the surgical process, and affect intraoperative monitoring, and postoperative hiccup may affect would healing and hemodynamic stability. Hiccup can lead to have increased aspiration risk. Hiccup are is an incompletely understood phenomenon with multiple etiologies. Intraoperative hiccup related to laryngeal mask airway placement has been reported, and it presents unique challenges in diagnosis and management. Both pharmacological and non-pharmacological interventions have been utilized with various level of success. All treatment strategies are primarily aimed at interrupting the hiccup reflex arc.

## I. Introduction

The medical term for hiccup is singultus. Hiccup is a relatively common phenomenon that arise abruptly and is generally short lived. It can affect both male and female in all age groups from infants to senior adults [1,2,3]. Fetal hiccups have also been interestingly observed relatively recently in utero during maternal ultrasonogram, and fetal hiccup is believed to help in respiratory efforts after delivery [1]. The duration of hiccup varies and, in most cases, it resolves spontaneously but can last significantly longer [4,5]. Based on the duration of hiccups, it may be classified as acute attacks if less than 48 hours, persistent (protracted) hiccups if more than 48 hours, and intractable hiccups if more than 1 month according to Steger, et al. [1,6]. or more than 2 months based on Chang’s definition [7]. When hiccups last for significantly longer duration, they are usually indicative of other pathologic conditions including central nervous system (CNS) tumors, toxic metabolic etiologies or psychogenic [1,8]. Prolonged periods of hiccups, lasting from hours to days, can adversely affect quality of life, leading to physical exhaustion, weight loss from not being able to eat, and psychological suffering [8]. Hiccup can be a symptom secondary to many medical conditions, such as renal impairment, diabetes mellitus, electrolyte imbalances, gastroesophageal reflux disease (GERD), hiatal hernia and abdominal cancers [1,8]. Intraoperative hiccup is usually due to an acute etiology which triggers the hiccup reflex [1]. Although involved neurologic components for hiccup reflex seem to have been identified, the physiological function of hiccup remains essentially unknown. Hiccup has been interpreted as a primitive reflex in fetus preventing swallowing of amniotic fluid, an archaic gill ventilation pattern, a fetal preparation for independent breathing [8] or a programmed isometric inspiratory muscle exercise, which is believed to be useless after the neonatal period. Hiccup may be induced by stimulation or irritation along the reflex arc [9]. After all, hiccup is a poorly understood phenomenon with no clear explanation on why we get it. Intraoperative hiccups are largely related to the technique of anesthesia performed and use of laryngeal mask airways isa common cause of intraoperative hiccups [10].

Laryngeal mask airway as an airway device has gained tremendous popularity because it avoids some of the issues associated with endotracheal intubation, while at the same time laryngeal mask airway provides a relatively reliable airway in spontaneously breathing patients [11]. Insertion of an endotracheal tube requires laryngoscopy which is known to potentially traumatize upper airway and other structures, leading to sore throat, loss of voice, and other complications [12]. However, laryngeal mask airway can also cause some complications such as sore throat, dysphagia, airway trauma, and hoarseness of voice [13]. Laryngeal mask airway has also been reported to induce hiccup [10]. Although, laryngeal mask airway -associated hiccup appears to be not a very common complication, the incidence has been reported to be in the range of 1–5% [10]. We reviewed the recent literature and summarized what is new in this subject.

## II. Physiological Mechanism of Hiccup

**Figure 1:**
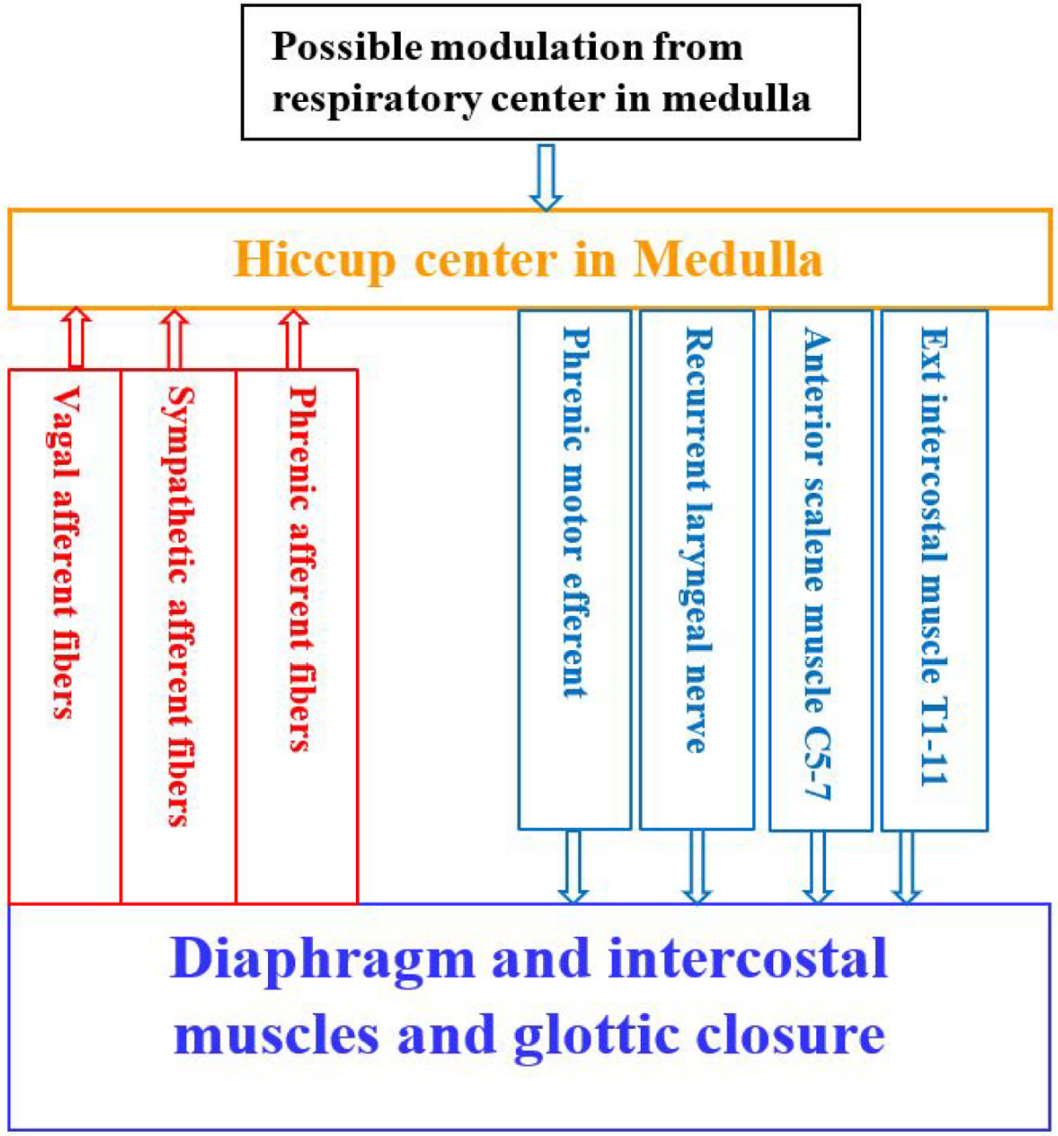
Proposed Hiccup reflex arc [1,6,15]. Hiccup center is located at Medulla. Afferent pathway includes vagal afferent fibers, sympathetic afferent fibers and phrenic afferent fibers. Efferent pathway includes External intercostal muscle T1–11, Anterior scalene muscle C5–7, Recurrent laryngeal nerve, and Phrenic motor efferent.

Hiccup is an involuntary myoclonic muscle contraction of the diaphragm and the intercostal muscles, which results in sudden inspiration and followed by abrupt closure of the glottis, generating the hic sound during hiccup [14]. Hiccup is believed to involve a reflex arc. The afferent limb consists of vagal, phrenic and sympathetic chain from T6–12. Impulses from the afferent arc travel to the upper medulla, specifically to the hiccup center. The hiccup center is located at the posterolateral part of the medulla oblongata of the brain stem. The efferent pathway travel in the motor fibers of the phrenic nerve to the diaphragm and accessory nerves to the inspiratory intercostal muscles. Stimulation anywhere along the afferent pathways may potentially lead to hiccups [15] [Figure 1]. Hiccups usually begin abruptly following a triggering stimulus and most often end abruptly or when the reflex arc has been interrupted. The relative ease of placing a laryngeal mask airway has made them a popular airway adjunct in the perioperative period. The distal end of a well seated laryngeal mask airway lies over the proximal esophagus, and sudden and rapid stretch of mechanoreceptors in the proximal esophagus is known to trigger the hiccup reflex [16]. Vagus nerve innervates the pharynx and upper esophagus and it is postulated that stimulation of the vagus nerve while insertion or rapid inflation of the laryngeal mask airway acts as a trigger for initiating hiccups. Some studies have shown more cases of hiccups with iGel insertion compared to other types of laryngeal mask airway but they were not statistically significant given the small number of cases involved. The use of positive pressure ventilation is yet another likely cause for hiccup. Nearly every patient after administration of anesthesia and muscle relaxant is placed on mechanical ventilation for the duration of the scheduled surgical procedure. Application of positive pressure to the airway can stimulate the respiratory system thereby inducing hiccup, and this was terminated with reduction of airway pressure [17].

Hiccup creates a pressure gradient across the lower esophageal sphincter (LES) enabling reflux and this could increase the risk for aspiration [18]. Thus, it becomes imperative that attempts be made to prevent aspiration risks from intraoperative hiccups. The pathophysiology of gastroesophageal reflux in most patients with GERD revolves around transient or lasting LES relaxation. Considering the pressure difference across LES is responsible for reflux after hiccup, it is also likely that patients with GERD might be at increased risk of developing hiccup. Patients with chronic hiccup, such as those with an underlying CNS or gastrointestinal (GI) issues may present as a challenge in the perioperative period. Even if not symptomatic at presentation, they remain at risk for aspiration, especially during induction [8]. These patients would more likely present with an acute episode after vagal stimulation or after diaphragmatic irritation from CO_2_ insufflation in laparoscopic procedures.

### III. Etiologies of Intraoperative Hiccup

1. Excessive food and carbonated beverages are among the many triggers that have been implicated as causes for hiccups [1,7].
2. Perioperative hiccup is more commonly induced by various anesthetic techniques and drugs used during anesthesia. Hiccup has been reported in patient receiving epidural anesthesia for vaginal hysterectomy, although this could also be due to excessive uterine stretching stimulating the hiccup reflex [18]. Epidural anesthesia seems less likely to be the cause for hiccups, because cervical epidural injection of local anesthetics has been used successfully to treat hiccup by blocking the peripheral vagal stimulation [5].
3. Some drugs used in anesthesia practice may induce hiccup. These drugs include methohexital, thiopentone, midazolam, and opioids [2,19]. Propofol has also been reported to be associated with hiccup, and being successfully treated with lidocaine [18]. While propofol is routinely used in all age groups, we don’t frequently encounter this complication probably because lidocaine is almost routinely administered prior to propofol to prevent pain on injection with propofol [20].
4. Bag mask ventilation prior to induction ensures pre-oxygenation and reduces the incidence of desaturation during intubation. Inadequate seal of the mask either due to poor technique or patient related factors can lead to inadvertent gastric insufflation. This can cause over distension of the stomach and is suggested to cause intraoperative hiccups [20].
5. Other drugs have also been indicated to cause hiccups. Dexamethasone is documented to cause hiccup [20]. Aripiprazole has also been reported to induce persistent hiccup. Aripiprazole is a psychoactive compound acting as a dopamine D_2_ partial agonist, serotonin 5-HT(1_A_) partial agonist and serotonin 5-HT(2_A_) antagonist [21].

Many patients scheduled for surgery do have prior diagnosis of GERD that does have anesthetic implications, because heartburn and regurgitation in patients with GERD are the most common presenting symptoms. GERD may present with hiccup and is reported in about 4.5 – 9.5% of the patients [22,23]. Anesthetic techniques and drugs reported to cause hiccup are summarized in Table 1.

## IV. Laryngeal Mask Airway-Related Hiccup

### 1. Incidence

The incidence of laryngeal mask airway -related hiccup is estimated to be around 5% [24,25]. In a study by Bapat, et al., the incidence of hiccups after laryngeal mask airway insertion differed with induction agent used. they reported an incidence of 2%, 4% and 14% with propofol, lidocaine with thiopentone and midazolam with thiopentone respectively [26].

### 2. Risk factors

Since the introduction of laryngeal mask airway back in 1981, various new models of supraglottic devices (SGAD) have been developed to ensure a safe and reliable airway. Several pre-existing conditions such as GERD can predispose a patient to hiccup in the intraoperative period. Different models of laryngeal mask airway may have different impact on the incidence of laryngeal mask airway -related hiccup. When laryngeal mask airway is compared to cuffed oropharyngeal airway (COPA), traditional laryngeal mask airway induces hiccup in 5.3% of the patients while COPA induces only 1.7% (P < 0.03%) [24]. When laryngeal mask airway is compared with ProSeal, traditional laryngeal mask airway induces hiccup in this study in 5.7% of the patients while ProSeal induces only 1.6% (P < 0.03%) [25]. When laryngeal mask airway is compared with iGel: The newer generation of SGADs like the iGel prioritizes reduction of aspiration risk with a drainage channel which accommodates suction of gastric contents, thus lowering the risk of aspiration. Several studies have attempted to investigate the difference between iGel and laryngeal mask airways in occurrence of hiccup as a complication. Patients who received iGel as an airway device compared with laryngeal mask airways were found to have a slightly higher incidence of hiccup although these were not found to be statistically significant [27]. There was also a study showing traditional laryngeal mask airway induces more gastric insufflation, though they did not show the incidence of hiccup [28].

Electrolyte imbalances such as hyponatremia, hypokalemia and hypocalcemia may present with hiccup. Renal impairment, particularly patients with symptoms of uremia [29]. These patients may present intraoperatively with hiccup although its association with chronic hiccup is well known. The external larynx lift technique was found to be potentially less likely to cause tissue trauma of the upper airway during laryngeal mask airway insertion [30].

## V. Diagnosis

The diagnosis of hiccup in daily life is generally not difficult due to its clear clinical presentations with a classic “hic” sound and an acute episode most often is self-limiting and terminates on its own. However, diagnosis of intraoperative hiccup may not be as easy. Since intraoperative hiccup may delay the beginning of surgery, interfere with the surgical process, and potentially affect intraoperative monitoring, prompt diagnosis and management are warranted. Intraoperative hiccup may not have the typical “hic” sound. But the abrupt myoclonic muscle contraction of the diaphragm and the intercostal muscles with the subsequent sudden inspiration movement can still indicate the occurrence of hiccup. Other clinical manifestations of intraoperative hiccup may include hemodynamic disturbances that include hypotension and bradycardia from negative intrathoracic pressure, although it is not very clear if this is clinically relevant [31]. A chronic episode is generally worked extensively, which includes a complete exam, blood work and medications patient might be taking.

## VI. Management

There is a lack of definitive guidelines on how to treat hiccup as there are many possible etiologies to go along with an incompletely understood reflex arc. Multiple anesthetic drugs and techniques used during anesthesia can induce an intraoperative attack of hiccup. And since the etiology is often multi-factorial, multiple modalities have been tried to treat hiccups (Table 2) [32]. Treatment is primarily aimed at interrupting the hiccup reflex arc and both pharmacological and non-pharmacological methods have been utilized targeting the phrenic and vagus nerve, the diaphragm and external intercostal muscles.

### 1. Pharmacological management

Various pharmacological agents have been used to treat acute hiccups in the intraoperative period, although the precise mechanism for the action is still speculative. It is now believed that there may be certain neurotransmitters involved that are responsible for triggering the reflex arc. The drugs that have been used to treat hiccups either decrease input from the periphery to the hiccup center or they decrease the excitatory impulse from the center [33]. Acetylcholine is one of many peripheral neurotransmitters that have been targeted successfully in treating hiccups. The effects of Acetylcholine on the GI tract increase the smooth muscle tone and contractility.

Anticholinergic agents like atropine (0.5 mg intravenously) have been shown to reduce intra-esophageal pressure [10]. Additionally, atropine can block not only the vagally-mediated afferent impulse from stimulation during laryngeal mask airway insertion and but also block the efferent arm from the hiccup center. Thus, atropine is believed to be an effective treatment of hiccups if laryngeal mask airway insertion was the most likely trigger.

Metoclopromide (10 mg intravenously) is used to treat hiccups through its antagonistic action on the dopamine receptor and serotonin agonism. It can be used orally or by intravenous route to treat hiccups although the IV route has proven to be more potent and quicker onset. Metoclopromide is considered one of the more reliable options for anesthetic induced hiccups [34]. In addition, by increasing LES pressure, metoclopramide can reduce the aspiration risk in these patients. We should be mindful of the extrapyramidal side effects from these agents [34]. It seems likely that the LES increase is due to anticholinergic property of metoclopramide on the GI tract as this effect can be reversed with atropine [35].

Midazolam has been commonly seen as a triggering agent for hiccup. However, in one study midazolam (5 mg intravenously) was shown to relieve hiccups almost immediately [36].

Proton Pump Inhibitors: as treatment of hiccups mainly revolves around identifying the triggers, patients with GERD would generally benefit from proton Pump inhibitors, which may include omeprazole, lansoprazole, dexlansoprazole, rabeprazole, and pantoprazole [37].

Chlorpromazine: despite the myriad of options available, yet chlorpromazine (25–50 mg intravenously) is the only FDA approved drug for treatment of hiccups, and it can be administered orally, intravenously or intramuscularly [38].

### 2. Non-pharmacological management

When the cause or trigger of hiccup is not known, it is prudent to attempt with non-pharmacological intervention. Since insufflating the stomach with air can be common during mask ventilation, deairing the stomach is something that could be helpful. The second-generation laryngeal mask airways like iGel are specially designed with a built-in draining channel. Placement of nasal airway lubricated with lidocaine as a treatment modality for hiccup has been known for a long time, and a recent article further highlights the utility of this age-old technique [39]. Stimulation of the pharynx opposite C2–3 is the proposed mechanism for terminating the hiccups after insertion of nasal airway [40,41]. Ventilatory strategies like lung recruitment and application of continuous positive airway pressure at 25–30 cm of H_2_O have also been described to treat hiccups intraoperatively [42]. Another technique that has been mentioned is a sub-occipital release technique where traction is applied to the posterior neck. This stretches the sub-occipital muscles in the C2 dermatome, thus reducing pressure on the vagus nerve and thereby eliminating hiccups [43]. Retention of CO_2_ by using paper bags have been known to interrupt hiccups. In the intraoperative setting, this can be achieved by deliberate hypoventilation during mechanical ventilation. It has been studied that ET_CO2_ must be at least 48 mmHg for hiccups to be stopped. High CO_2_ levels would oppose hiccup signals arising from the medulla [44]. Smelling some salts like ammonium chloride have also been described as a quick and easy way to terminate hiccup. This can be advantageous in spontaneously breathing patients and sedated patients undergoing procedures where patient movement may not be tolerated [45]. Stellate Ganglion Block in treatment of hiccups is a relatively newer technique that has been tried. The mechanism of action is unclear, likely due to its sympathetic blockade in the hiccup reflex arc [4]. Cervical epidural block has been reported for the management of postoperative intractable hiccups [5]. Acupuncture has also been successfully used in the management of hiccup in liver cancer patients [46] and patients after stroke [47].

## Summary

Hiccup is an abrupt involuntary contraction of the diaphragm and intercostal muscles leading to sudden inspiration and closure of the glottis. Hiccup is not very rare in normal humans and it has various etiologies. The presence of hiccup in the perioperative period can be problematic. Intraoperative hiccup can be caused by anesthetic techniques and/or anesthetic drugs, and other medications. laryngeal mask airway may cause intraoperative hiccup, the incidence is estimated to be around 1–5%. Intraoperative hiccup can be managed by pharmacologic agents which may include anticholinergic agents like atropine, metoclopromide, midazolam, proton pump inhibitors, and chlorpromazine. It can also be managed non-pharmacologically by the stomach deairing, nasal airway lubricated with lidocaine, maintaining continuous positive airway pressure at 25–30 cm of H_2_O, sub-occipital release technique, and stellate Ganglion Block. All treatment strategies are primarily targeting on interrupting the hiccup reflex arc.

## Figures and Tables

**Table 1:** Anesthetic techniques and drugs that induce intraoperative hiccups.

Anesthetic techniques	Anesthetic drugs	Other drugs
Epidural	Methohexital	Aripiprazole
Mask ventilation	Thiopentone	dexamethasone
	Midazolam	
	Opioids	
	Propofol	

**Table 2:** Management of intraoperative hiccup.

Pharmacological management	Non-Pharmacological management
Anticholinergic agents like atropine	Deairing the stomach
Metoclopromide	Nasal airway lubricated with lidocaine
Midazolam	CPAP at 25–30 cm of H_2_O
Proton Pump Inhibitors	Sub-occipital release technique
Chlorpromazine	Stellate Ganglion Block
Nifedipine	Acupuncture

**CPAP:** Continuous positive airway pressure.
